# Transcriptomic and Metabolomic Analyses of the Effects of Exogenous Trehalose on Heat Tolerance in Wheat

**DOI:** 10.3390/ijms23095194

**Published:** 2022-05-06

**Authors:** Yin Luo, Yue Wang, Yanyang Xie, Yamin Gao, Weiqiang Li, Shuping Lang

**Affiliations:** 1 Instrument Sharing Platform of School of Life Sciences, East China Normal University, Shanghai 200241, China; wangyue1998sh@ccmail.win (Y.W.); xieyanyang0318@163.com (Y.X.); ymgao@bio.ecnu.edu.cn (Y.G.); 2 Key Laboratory of Mollisols Agroecology, Northeast Institute of Geography and Agroecology, Chinese Academy of Sciences, Changchun 130102, China; weiqiangli@henu.edu.cn; 3 Jia Xing Academy of Agricultural Science, Jiaxing 314016, China; littlelsp@163.com

**Keywords:** trehalose, wheat, heat stress, transcriptome, metabolome

## Abstract

Trehalose can improve the tolerance of plants to various types of environmental stress. Nonetheless, information respecting the molecular networks of wheat seedlings to exogenous trehalose under heat stress is limited. Here, two wheat varieties pretreated with exogenous trehalose were selected to explore the molecular mechanism by which trehalose improves the heat tolerance of wheat (*Triticum aestivum* L.). The results indicated that exogenous trehalose improved the physiological state of wheat seedlings under heat stress. Through RNA sequencing and metabolomics analysis, the genes and metabolites specifically expressed in trehalose pretreatment were identified. After heat stress, there were 18,352 differentially expressed genes (DEGs) in the control and trehalose-treated (H_vs_TreH) groups of Yangmai 18 and 9045 DEGs in Yannong 19. Functional annotation and enrichment analyses showed that the DEGs in the two wheat varieties were mainly involved in carbohydrate metabolism and biosynthesis of secondary metabolites. Through a liquid chromatography–mass spectrometry platform, 183 differential metabolites in H_vs_TreH groups of Yangmai 18 and 77 differential metabolites in Yannong 19 were identified. Compared with the control group, many protective metabolites, such as amino acids, purines, phenylpropanoids and flavonoids, showed significant differences under heat stress. The results indicated that exogenous trehalose protected the wheat biomembrane system, enhanced carbohydrate metabolism and signal transduction, strengthened the activity of the tricarboxylic acid cycle (TCA cycle), regulated purine metabolism, gene expression and metabolite accumulation in the phenylpropanoid biosynthesis and flavonoid biosynthesis pathways, thus improving the heat tolerance of wheat.

## 1. Introduction

The global surface temperature is expected to rise by 0.8–4.8 °C by the end of the 21st century [1]. Rising temperature negatively affects plants, such as by destroying the ultrastructure of the chloroplast and thylakoid membranes, decreasing the photosynthetic rate [2,3] and oxidative phosphorylation efficiency [4], inducing leaf senescence, destroying the reproductive development system, and reducing crop yield [5]. In response to heat stress, plants have evolved complex adaptation mechanisms, such as leaf cooling, molecular chaperones [6], and the accumulation of osmoregulatory substances (such as betaine, proline and trehalose), which may come at the cost of reducing growth and productivity at the same time [7].

As a non-reducing disaccharide, trehalose (α-D-glucopyranosyl α-D-glucopyranoside) is widely found in various organisms. In plants, trehalose-6-phosphate synthase (TPS) helps to synthesize trehalose-6-phosphate (T6P) from a glucose-6-phosphate and uracil diphosphate-glucose, and then the phosphate group of T6P is removed by trehalose-6-phosphate phosphatase (TPP) to produce trehalose, which is ultimately degraded by trehalase to form glucose.

Exogenous application of trehalose has great potential in improving abiotic and biotic stress resistance, and several mechanisms are involved in this process. Under nitrogen-restriction conditions, trehalose spraying on tobacco (*Nicotiana tabacum*) leaves can alleviate the symptoms of nitrogen deficiency by upregulating nitrate and ammonia assimilation related genes and increasing the activities of relevant enzymes [8]. Under drought stress, exogenous application of trehalose to maize (*Zea mays* L.) enhanced antioxidant activity with increases in oil tocopherol, total flavonoid, and total phenolic contents [9]. Trehalose accumulation is also considered to be a fundamental strategy in the process of restoring plant stress resistance [10], which can indirectly eliminate reactive oxygen species (ROS) in vivo by maintaining the function of biomembranes and proteins [2,11] or affecting hormone synthesis [12]. Currently, the systematic mechanism is still unclear. Transcriptome data show that many trehalose biosynthesis genes have obvious responses to drought, salt, and cold stress in both roots and branches [13]. However, overexpression of the endogenous trehalose synthase gene significantly affected trehalose accumulation, which was associated with increased trehalase activity, indicating that trehalose could not directly act as a protector or stabilizer of cellular structures [14]. In addition, it is suggested that the trehalose intermediate T6P may act as a sugar signal and cooperate with other substances such as sucrose, sucrose nonfermenting 1-related protein kinase 1 (SnRK1) and hormones to respond to abiotic stress [15]. Though Shi et al. [16] have used exogenous trehalose to improve the resistance of tobacco to tobacco mosaic disease and defined the trehalose response gene network through transcriptome analysis, the molecular regulatory network and signal transduction mechanism of exogenous trehalose in response to heat stress have not yet been reported.

As one of the three major food crops, wheat (*Triticum aestivum* L.) is sensitive to high-temperature stress. On the basis of a global scale model, for every 1 °C increase in global mean annual temperature, global wheat production tends to decline by 4.1–6.4% by the middle of the 21st century [17,18]. To avoid serious losses in agriculture and the food industry, it is of far-reaching significance to explore the heat tolerance of wheat. In this study, transcriptome and metabolomics techniques were used to jointly analyze the mechanisms of exogenous trehalose to improve the heat tolerance of wheat.

## 2. Results

### 2.1. Effects of Exogenous Trehalose on Phenotype and Physiological Indexes of Two Wheat Varieties under Heat Stress

**Figure 1:**
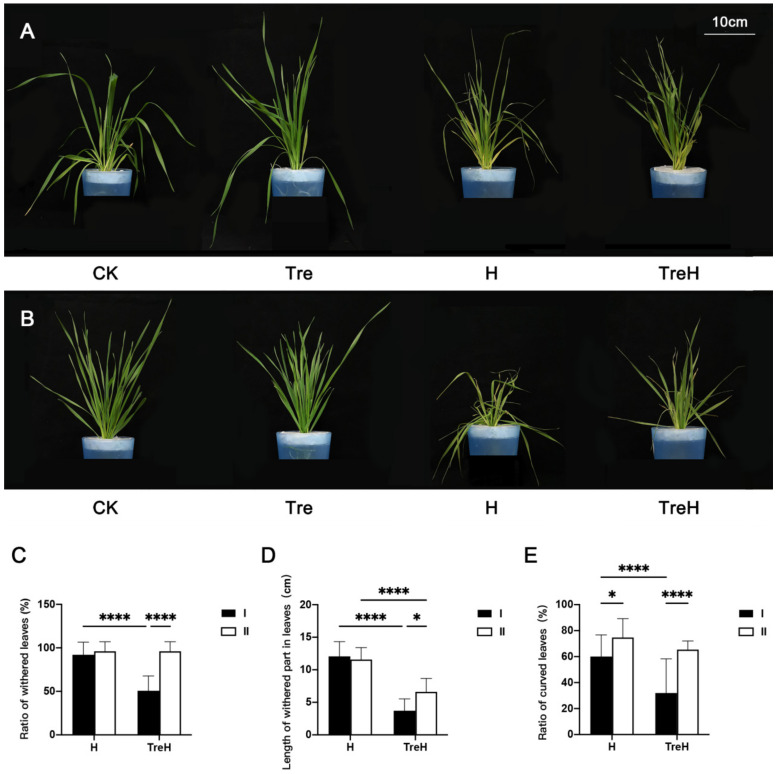
Phenotypic changes of wheat induced by exogenous trehalose under heat stress. (**A**) Yangmai 18. (**B**) Yannong 19. CK—control group; H—heat stress treatment for 24 h; Tre—trehalose pretreatment for 3 d; TreH—trehalose pretreatment for 3 d followed by high-temperature treatment for 24 h. A total of plants were randomly selected from each treatment group for taking photos. All the photos were taken soon after the high-temperature groups finished the heat-stress treatment. (**C**) The ratio of withered leaves in wheat seedlings. (**D**) Length of withered part in leaves. (**E**) The ratio of curved leaves in wheat seedlings. I—Yangmai 18; II—Yannong 19; H—heat stress treatment for 24 h; TreH—trehalose pretreatment for 3 d followed by high-temperature treatment for 24 h. Values are the means ± SDs (*n* = 3). * indicates a significant difference at 0.01 < *p* ≤ 0.05, **** indicates a significant difference at *p* ≤ 0.0001.

Leaves of two wheat varieties wilted and turned yellow under high-temperature stress (Figure 1A,B). Compared with plants without trehalose pretreatment under high-temperature stress, the trehalose pretreatment group showed a reduced degree of leaf wilting and yellowing caused by heat stress (Figure 1A,B). Following high temperature, the trehalose pretreatment group also indicated a lower ratio of withered leaves, length of withered part in leaves and ratio of curved leaves, especially in Yangmai 18 (Figure 1C–E).

**Figure 2:**
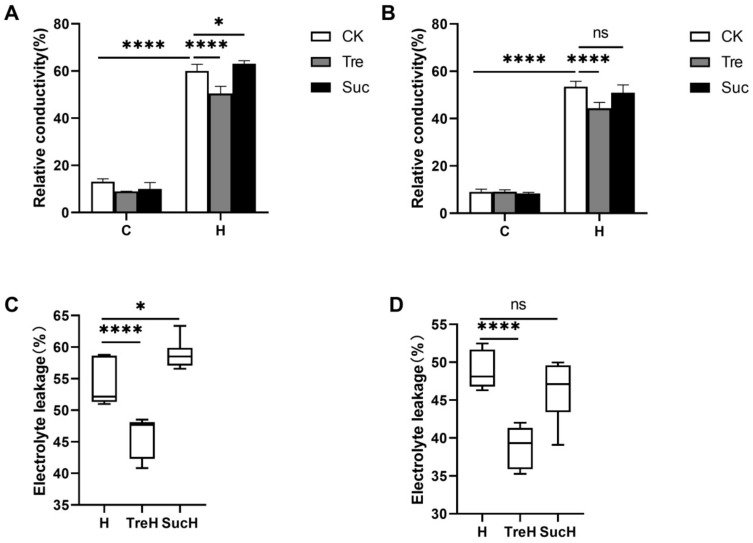
The relative electrical conductivity and electrolyte leakage of two varieties of wheat seedlings. (**A**,**B**) C—room temperature; H—high temperature; CK—control group; Tre—trehalose pretreatment group; Suc—sucrose pretreatment group; (**C**,**D**) H—high-temperature stress treatment for 24 h; TreH—trehalose pretreatment for 3 d followed by high temperature stress treatment for 24 h; SucH—sucrose pretreatment for 3 d followed by high temperature stress treatment for 24 h. (**A**,**C**) Yangmai 18. (**B**,**D**) Yannong 19. Values are the means ± SDs (*n* = 3). * indicates a significant difference at 0.01 < *p* ≤ 0.05, **** indicates a significant difference at *p* ≤ 0.0001, ns indicates no statistical difference.

Relative conductivity and electrolyte leakage (EL) are commonly used to indicate the degree of damage to the cell membrane and the level of stress resistance. Under high-temperature stress, compared with the control, the relative conductivity of Yangmai 18 and Yannong 19 increased significantly at high temperatures, indicating that the cell membrane was damaged by heat stress (Figure 2A,B). After the application of trehalose, the relative conductivity and EL in the two varieties of wheat seedlings decreased significantly, indicating that exogenous trehalose played an important role in alleviating the peroxidation damage of wheat membrane lipid caused by high-temperature stress (Figure 2). In addition, in order to determine the specificity of trehalose, sucrose similar to trehalose was selected as the analogue of trehalose for control treatment. The results showed that under high-temperature stress, the relative conductivity of wheat leaves after sucrose treatment was not different from that of the high-temperature treatment group (Figure 2). These results indicated that trehalose treatment could maintain membrane stability to offset the effects of high-temperature stress.

### 2.2. Effects of Exogenous Trehalose on Chloroplast Ultrastructure of Two Wheat Varieties under Heat Stress

**Figure 3:**
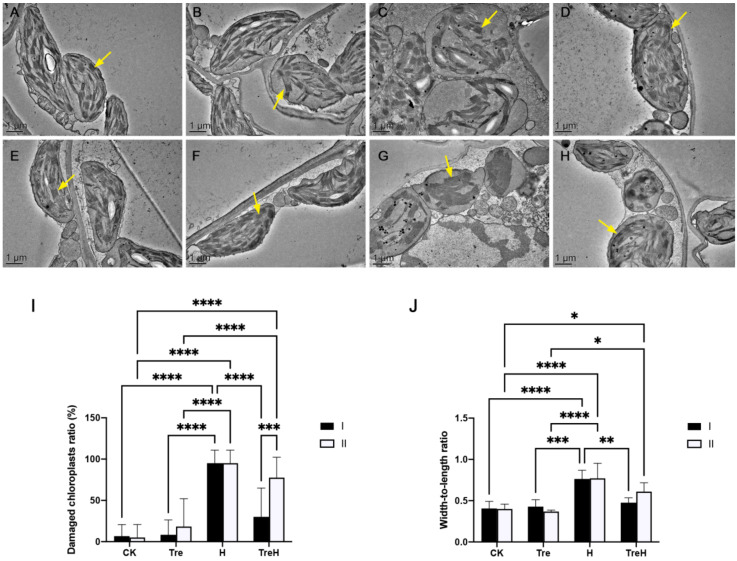
Chloroplast ultrastructure of two varieties of wheat (×2500). (**A**) Yangmai 18 without trehalose at room temperature. (**B**) Yangmai 18 trehalose pretreatment group. (**C**) Yangmai 18 without trehalose at high temperature. (**D**) Yangmai 18 high temperature trehalose pretreatment group. (**E**) Yannong 19 without trehalose at room temperature. (**F**) Yannong 19 trehalose pretreatment group. (**G**) Yannong 19 without trehalose at high temperature. (**H**) Yannong 19 high-temperature trehalose pretreatment group. Yellow arrow—thylakoid. The images were taken by a transmission electron microscope. Scale bars = 1 μm. (**I**) Damaged chloroplasts ratio. (**J**) Width-to-length ratio of chloroplasts. I—Yangmai 18; II—Yannong 19; CK—control group; H—heat stress treatment for 24 h; Tre—trehalose pretreatment for 3 d; TreH—trehalose pretreatment for 3 d followed by high-temperature treatment for 24 h. Values are the means ± SDs (*n* = 3). * indicates a significant difference at 0.01 < *p* ≤ 0.05, ** indicates a significant difference at *p* ≤ 0.01, *** indicates a significant difference at *p* ≤ 0.001, **** indicates a significant difference at *p* ≤ 0.0001.

At room temperature, chloroplasts of mesophyll cells were distributed at the edge of cells, close to the cell wall, and were spindle-shaped, full in shape and complete in structure. At the same time, grana lamellae were orderly and parallel, thylakoid lamellae were orderly and the membrane structure was complete (Figure 3A,E). The chloroplast morphology of the trehalose-pretreated group at room temperature was not much different to that of the untreated trehalose group at room temperature (Figure 3B,F). Following high-temperature treatment, chloroplasts began to expand and showed an oval shape. Most chloroplasts were still distributed at the edges of cells but began to break away from the cell wall. Meanwhile, the chloroplast membrane no longer clung to the thylakoid structure, and the granum lamellae structure was loose, deformed and broken. In Yangmai 18, the chloroplast membrane was severely broken, and the granum lamellae were disordered (Figure 3C). Pretreatment with trehalose showed a good protective effect because the membrane structure was intact and the granum lamellae were orderly (Figure 3D). In Yannong 19, the protective effect of trehalose pretreatment was not as obvious as that in Yangmai 18. The granum lamellae structure was clear but began to deform, some chloroplasts were still spindle-shaped, and the membrane structure was complete (Figure 3G,H). Compared with plants without trehalose pretreatment under high-temperature stress, the trehalose pretreatment group reduced the proportion of damaged chloroplasts in leaves (Figure 3I). Similarly, the width-to-length ratio of chloroplasts decreased in the trehalose pretreatment group, and this heat-resistant effect is more significant in Yangmai 18 (Figure 3J).

### 2.3. RNA Sequencing Data Quality Evaluation

The results of sequencing data showed that the GC contents of all samples were over 52%, and the average base mass of Q30 was over 94% (Supplementary Table S3), indicating that the transcriptome sequencing was of good quality and met the requirements of data analysis. Taking Chinese Spring as the reference genome for comparison, the comparison rate between each sample and the reference genome was over 93% (Supplementary Table S3), and the read ratio of the reference genome in the unique comparison of each sequenced sample was over 88%, indicating that the comparison results of the selected reference genes could meet the requirements of gene function annotation and analysis.

**Figure 4:**
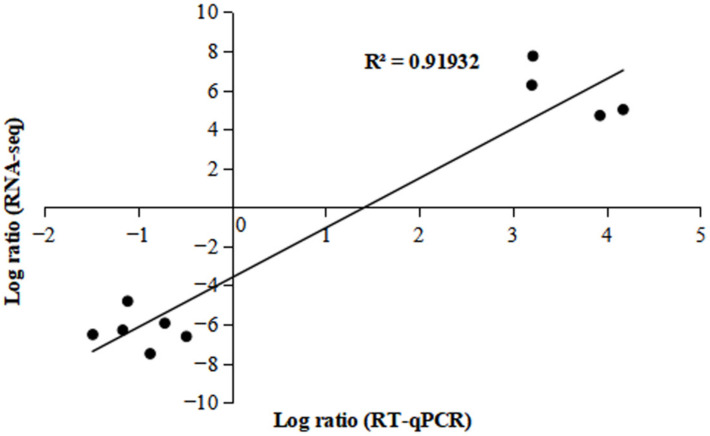
Validation of transcriptomic data (10 DEGs) RT-qPCR. Each black circle represents a gene.

To validate the mRNA sequencing data, 10 DEGs identified as trehalose-responsive candidates were selected and analyzed by real-time quantitative PCR (RT-qPCR) (Figure 4 and Supplementary Table S4). As shown in Figure 4, the change trend of gene expression relative to fold change (FC) in transcriptome data was the same as that in RT-qPCR data, indicating the reliability of RNA-seq.

### 2.4. Statistical Analysis

**Figure 5:**
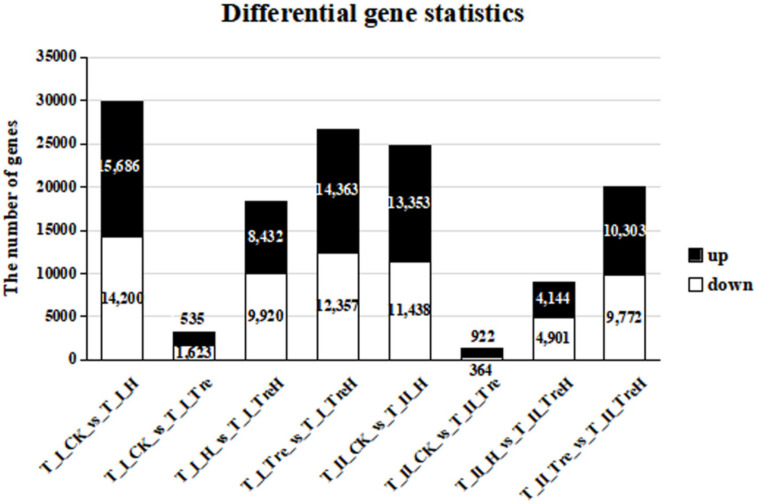
Differential gene statistics. T—the transcriptome; Ⅰ—Yangmai 18; Ⅱ—Yannong 19; CK—control group; H—heat stress treatment for 24 h; Tre—trehalose pretreatment for 3 d; TreH—trehalose pretreatment for 3 d followed by high-temperature treatment for 24 h.

According to |log_2_ FC| ≥ 1 and false discovery rate (FDR) < 0.05, the total numbers of differential genes, upregulated genes and downregulated genes were screened out (Table 1 and Figure 5). Among them, the T_I_CK_vs_T_I_H and T_I_Tre_vs_T_I_TreH groups had more differential expressed genes, which indicated that Yangmai 18 was more sensitive and more responsive than Yannong 19 when suffering from heat stress. However, without heat stress, trehalose had relatively little effect on gene expression in wheat (T_II_CK_vs_T_II_Tre and T_I_CK_vs_T_I_Tre).

### 2.5. Gene Ontology (GO) Function Analysis of DEGs

**Figure 6:**
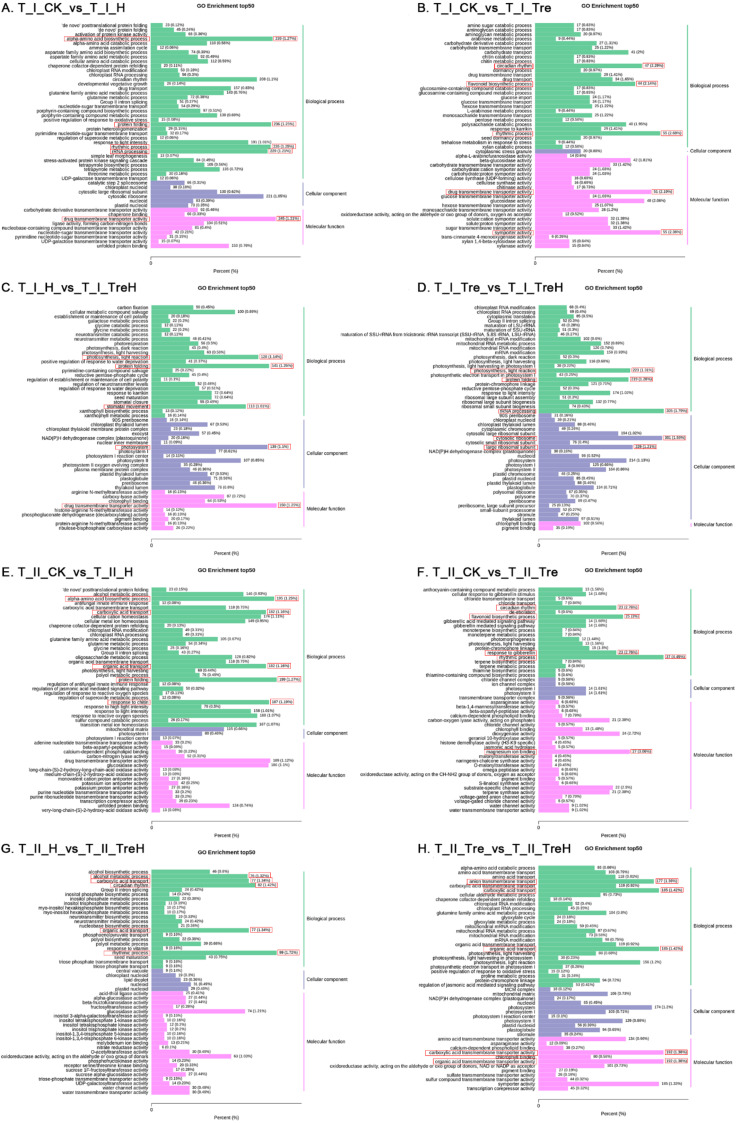
GO enrichment of the DEGs. T—the transcriptome; I—Yangmai 18; II—Yannong 19; CK—control group; H—heat stress treatment for 24 h; Tre—trehalose pretreatment for 3 d; TreH—trehalose pretreatment for 3 d followed by high-temperature treatment for 24 h. The abscissa represents the ratio of the genes annotated to the entry to the total number of annotated genes, and the ordinate represents the name of the GO entry. The label on the right side of the graph represents the category to which the GO entry belongs. The red box represents the top 5 DEG ratios in the figure.

To further analyze the function of DEGs, GO-enrichment analysis of DEGs was performed in each treatment group. When Yangmai 18 was exposed to heat stress, the group without trehalose (T_I_CK_vs_T_I_H) showed that the functions of DEGs were mainly concentrated in the amino acid biosynthetic process, protein folding, rhythm process, rRNA processing, and drug transmembrane transport activity (Figure 6A). After trehalose was applied (T_I_Tre_vs_T_I_TreH), the functions of DEGs in the heat-stress group and nonstress groups were mainly concentrated in photosynthesis, light reaction, protein folding, rRNA processing, cytosolic ribosomes and large ribosomal subunits (Figure 6D).

The T_II_CK_vs_T_II_H group showed that when Yannong 19 wheat encountered heat stress, the functions of DEGs were mainly concentrated in the amino acid biosynthesis process, carboxylic acid transport, organic acid transport, protein folding, and response to chitin (Figure 6E). However, when trehalose was applied, the functions of the DEGs (T_II_Tre_vs_T_II_TreH) were mainly concentrated in carboxylic acid transport, carboxylic acid transmembrane transporter activity, organic acid transport, organic acid transmembrane transporter activity and anion transmembrane transport (Figure 6H).

Under room temperature conditions, the functions of DEGs in Yangmai 18 (T_I_CK_vs_T_I_Tre) were mainly concentrated in circadian rhythm, rhythmic process, flavonoid biosynthetic process, drug transmembrane transport and symporter activity (Figure 6B) and those in Yannong 19 (T_II_CK_vs_T_II_Tre) were mainly concentrated in circadian rhythm, flavonoid biosynthetic process, rhythm process, response to gibberellin, and magnesium ion binding (Figure 6F).

Under high temperature, the functions of DEGs in Yangmai 18 (T_I_H_vs_T_I_TreH) were mainly concentrated in photosynthesis, light reaction, protein folding, stomatal movement, photosystem and drug transmembrane transporter activity (Figure 6C), while those in Yannong 19 (T_II_H_vs_T_II_TreH) were mainly concentrated in alcohol metabolic process, carboxylic acid transport, circadian rhythm, organic acid transport, and rhythm process (Figure 6G).

### 2.6. Enrichment of DEGs by Kyoto Encyclopedia of Genes and Genomes (KEGG) Metabolic Pathway

**Figure 7:**
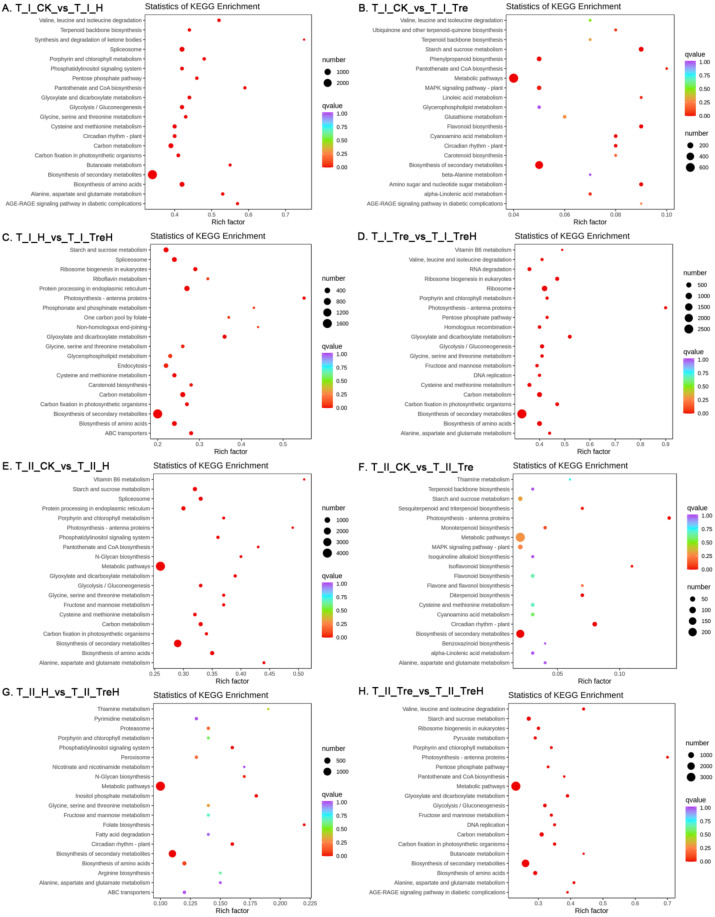
KEGG enrichment of the DEGs. T—the transcriptome; I—Yangmai 18; II—Yannong 19; CK—control group; H—heat stress treatment for 24 h; Tre—trehalose pretreatment for 3 d; TreH—trehalose pretreatment for 3 d followed by high-temperature treatment for 24 h. The ordinate represents the KEGG pathway. The abscissa represents the rich factor. The larger the rich factor, the greater the enrichment degree. The larger the dot, the more the number of differential genes enriched by the pathway. The redder the color of the dot, the more significant the enrichment.

To explore the metabolic regulatory networks of the two wheat varieties after trehalose pretreatment under heat stress, the KEGG metabolic pathways of the DEGs in each group were analyzed. When Yangmai 18 was exposed to heat stress, the DEGs in the group without trehalose pretreatment (T_I_CK_vs_T_I_H) were mainly concentrated in spliceosome, carbon metabolism, biosynthesis of amino acids, glycolysis/glyconeogenesis, cysteine and methionine metabolism (Figure 7A). At room temperature, the DEGs in Yangmai 18 (T_I_CK_vs_T_I_Tre) without trehalose pretreatment were mainly concentrated in metabolic pathways, biosynthesis of secondary metabolites, starch and sucrose metabolism, phenylpropanoid biosynthesis and MAPK signaling pathway-plant (Figure 7B). At high temperature, DEGs in the Yangmai 18 group (T_I_H_vs_T_I_TreH) without trehalose pretreatment were mainly concentrated in metabolic pathways such as starch and sucrose metabolism, spliceosome, protein processing in endoplasmic reticulum, carbon metabolism and biosynthesis of amino acids (Figure 7C). At high temperature, after trehalose pretreatment (T_I_Tre_vs_T_I_TreH), DEGs were mainly concentrated in metabolic pathways such as biosynthesis of secondary metabolites, ribosomes, carbon metabolism and biosynthesis of amino acids (Figure 7D).

In the absence of trehalose, the DEGs in Yannong 19 in the room temperature and high-temperature groups (T_II_CK_vs_T_II_H) were mainly concentrated in metabolic pathways, biosynthesis of secondary metabolites, starch and sucrose metabolism, protein processing in the endoplasmic reticulum and biosynthesis of amino acids (Figure 7E). Under room temperature conditions, DEGs in Yannong 19 (T_II_CK_vs_T_II_Tre) without trehalose pretreatment were mainly concentrated in the biosynthesis of secondary metabolites and circadian rhythm plants (Figure 7F). Under high temperature, DEGs in the nontrehalose and trehalose-treated Yannong 19 groups (T_II_H_vs_T_II_TreH) were mainly concentrated in metabolic pathways, biosynthesis of amino acids, circadian rhythm plants, phosphatidylinositol signaling systems and inositol phosphate metabolism (Figure 7G). After trehalose was applied, the DEGs in Yannong 19 in the room-temperature and high-temperature groups (T_II_Tre_vs_T_II_TreH) were mainly concentrated in metabolic pathways and biosynthesis of secondary metabolites (Figure 7H).

KEGG enrichment analysis showed that heat stress mainly affected the biosynthesis of amino acids in wheat, but the metabolic pathways of DEG enrichment between the two wheat varieties were quite different, which may be related to the different characteristics of the varieties.

### 2.7. Analysis of Trehalose-Responsive Candidate Genes and Transcription Factors

**Figure 8:**
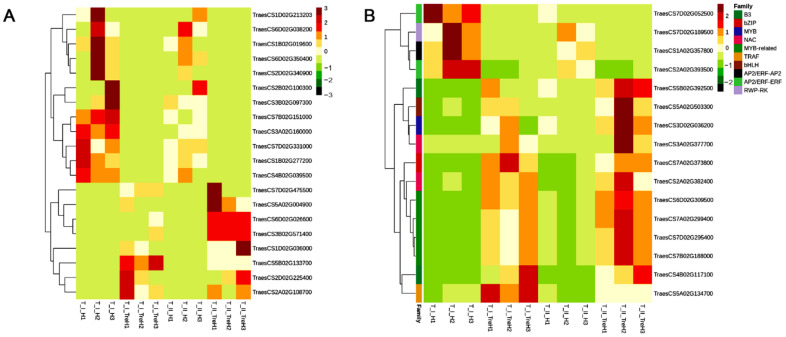
Heatmap of trehalose response candidate genes and transcription factors. T—the transcriptome; I—Yangmai 18; II—Yannong 19; CK—control group; H—heat stress treatment for 24 h; Tre—trehalose pretreatment for 3 d; TreH—trehalose pretreatment for 3 d followed by high-temperature treatment for 24 h. (**A**) Heatmap of trehalose response candidate genes. The abscissa represents the sample name and the ordinate represents the ID of genes. Red indicates high expression and green indicates low expression. (**B**) Heatmap of trehalose response candidate transcription factors. The abscissa represents the sample name, and the ordinate represents the ID of transcription factor genes and their families. Red indicates high expression and green indicates low expression.

According to FC, the DEGs in the T_I_H_vs_T_I_TreH and T_II_H_vs_T_II_TreH groups were analyzed, and the trehalose-responsive candidate genes in the two varieties were screened out. A total of 8 upregulated genes and 12 downregulated genes were found, which may respond to trehalose signals to improve the heat tolerance of wheat under high-temperature stress (Figure 8A). According to the gene function annotations, these genes can be divided into six categories: genes related to plant pathogen defense (TraesCS5B02G133700, TraesCS3B02G571400, TraesCS5A02G356800), apoptosis (TraesCS7D02G475500, TraesCS2D02G225400, TraesCS4B02G039500), lipid metabolism (TraesCS5A02G004900, TraesCS1D02G036000, TraesCS5A02G004800), plant hormones (TraesCS7B02G151000, TraesCS1B02G019600), protein ubiquitination-related genes (TraesCS7D02G331000, TraesCS1D02G213203), and other genes.

Transcription factors also play an important role in the trehalose response. The DEGs in T_I_H_vs_T_I_TreH and T_II_H_vs_T_II_TreH were analyzed according to FC, and 12 upregulated genes and 4 downregulated genes were identified that may respond to trehalose signals to improve the heat tolerance of wheat under heat stress (Figure 8B). Among them, genes of B3, bZIP, MYB, NAC, MYB-related, TRAF, and bHLH were upregulated, while AP2/ERF-AP2, AP2/ERF-ERF and RWP-RK were downregulated.

### 2.8. Differential Metabolite Analysis

**Figure 9:**
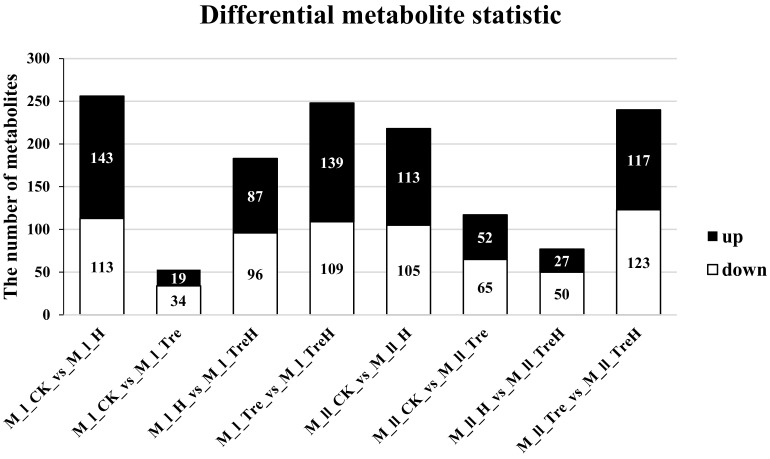
Differential metabolite statistics. M—the metabolomics; I—Yangmai 18; II—Yannong 19; CK—control group; H—heat stress treatment for 24 h; Tre—trehalose pretreatment for 3 d; TreH—trehalose pretreatment for 3 d followed by high-temperature treatment for 24 h.

After high-temperature stress, the metabolites of the two varieties of wheat had different response mechanisms. In this experiment, metabolites with FC ≥ 1.5 and FC ≤ 0.67 were selected, in which those with variable important in projection (VIP) ≥ 1 were selected as differential metabolites, and the total numbers of differential metabolites, upregulated metabolites and downregulated metabolites in each group were counted (Table 2 and Figure 9). Among them, the M_l_CK_vs_M_l_H group had more differential metabolites, while the M_l_CK_vs_M_l_Tre and M_ll_H_vs_M_ll_TreH groups had fewer differential metabolites, indicating that high temperature and exogenous trehalose might have a greater influence on the metabolite regulation of Yangmai 18 (Figure 9).

### 2.9. Enrichment Analysis of KEGG Metabolic Pathways of Differential Metabolites

**Figure 10:**
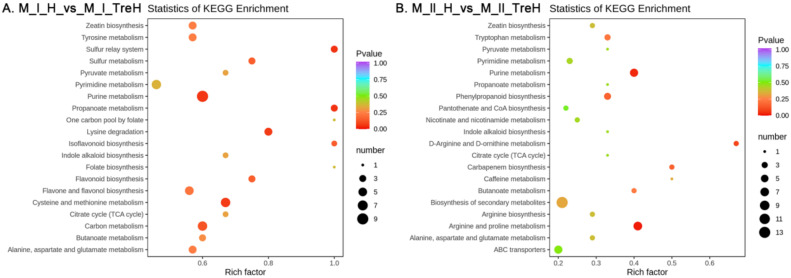
KEGG enrichment analysis of differential metabolites. M—the metabolomics; I—Yangmai 18; II—Yannong 19; H—heat stress treatment for 24 h; TreH—trehalose pretreatment for 3 d followed by high-temperature treatment for 24 h. The ordinate represents the KEGG pathway. The abscissa represents the rich factor. The larger the rich factor, the greater the enrichment degree.

According to the results of differential metabolites, KEGG pathway enrichment was carried out, and it was found that differential metabolites in the M_l_H_vs_M_l_TreH group were mainly enriched in purine metabolism, cysteine and methionine metabolism, carbon metabolism, flavone and flavonol biosynthesis and lysine degradation (Figure 10A). Differential metabolites in the M_ll_H_vs_M_ll_TreH group were mainly concentrated in purine metabolism, biosynthesis of secondary metabolites, arginine and proline metabolism, tryptophan metabolism and phenylpropanoid biosynthesis (Figure 10B). KEGG enrichment analysis revealed that trehalose mainly affected the content of amino acid-related metabolites in wheat under heat stress, and the metabolites of purine metabolic pathway in two varieties of wheat were enriched more, with more significant differences.

### 2.10. Integrated Analysis of DEGs and Differential Metabolites

**Figure 11:**
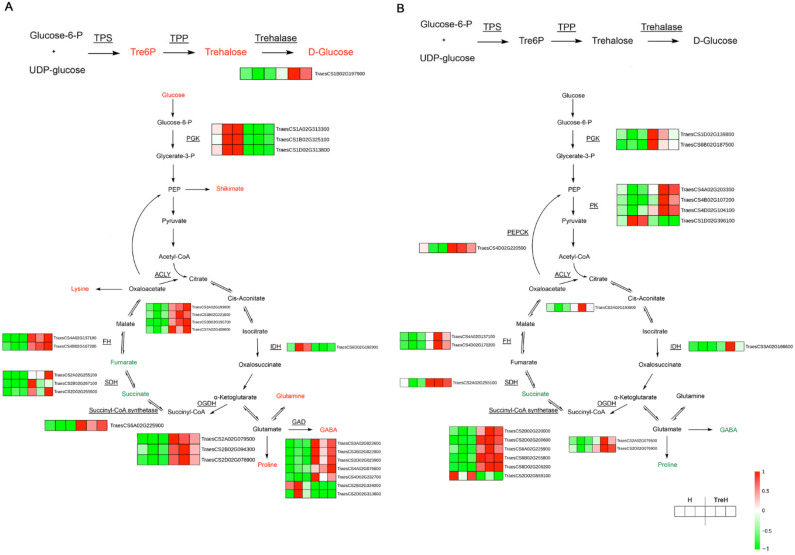
Gene-metabolite integrated analysis network of trehalose, amino acid and tricarboxylic acid cycle (TCA cycle) metabolic pathways. (**A**) Map of Yangmai 18. (**B**) Map of Yannong 19. The red words represent the metabolites upregulated and the green words represent the metabolites downregulated. The underlined genes were differentially expressed and their relative expression levels (FPKM value) in two groups (three biological replicates of each treatment) were shown as heat maps. H—heat stress treatment for 24 h; TreH—trehalose pretreatment for 3 d followed by high-temperature treatment for 24 h. Abbreviations: UDP-glucose, uracil diphosphate-glucose; TPS—trehalose-6-phosphate synthase; TPP—trehalose-6-phosphate phosphatase; PGK—phosphoglycerate kinase; PEP—phosphoenolpyruvate; ACLY—ATP citrate (pro-S)-lyase; IDH—isocitrate dehydrogenase; OGDH—2-oxoglutarate dehydrogenase E1 component; SDH—succinate dehydrogenase; FH—fumarate hydratase; GAD—glutamate decarboxylase; GABA—γ-aminobutyric acid; PEPCK—phosphoenolpyruvate carboxykinase; PK—pyruvate kinase.

In Yangmai 18, DEGs were significantly enriched in starch and sucrose metabolism pathways after trehalose pretreatment of wheat seedlings under heat stress, which indicated that exogenous trehalose promoted sugar metabolism and provided an energy source for wheat during heat stress. We constructed a gene-metabolite network using RNA-seq and metabonomics data (Figure 11A). Genes and metabolites involved in the metabolism of carbohydrates (such as T6P, glucose, and trehalose), amino acids (such as glutamine, lysine, and proline) and TCA cycles (such as isocitrate dehydrogenase (IDH), succinate dehydrogenase (SDH), and oxoglutarate dehydrogenase (OGDH)) had significant regulatory effects under the action of exogenous trehalose, which indicated their potential role in responding to trehalose signals and coping with heat stress. In addition, many genes related to guanine, xanthine, and adenosine synthesis were upregulated in purine metabolism, which indicated that trehalose also greatly affected this pathway at high temperature (Supplementary Figure S3A).

**Figure 12:**
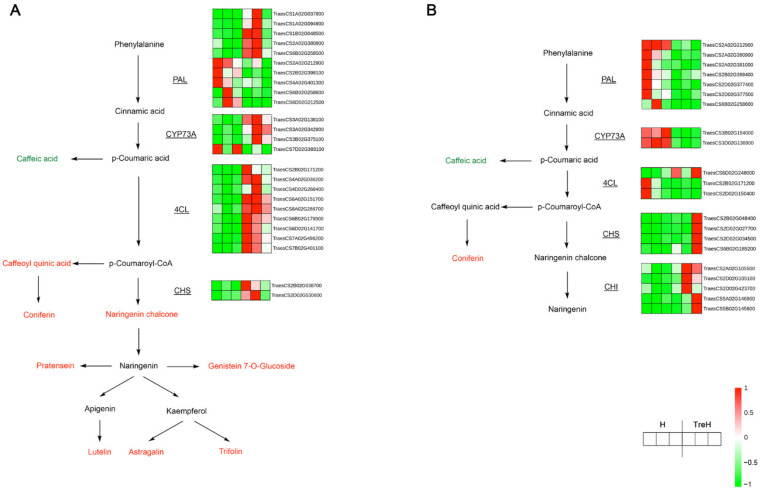
Gene-metabolite integrated analysis network of phenylpropanoid and flavonoid biosynthesis pathways. (**A**) Map of Yangmai 18. (**B**) Map of Yannong 19. The red words represent the metabolites upregulated and the green words represent the metabolites downregulated. The underlined genes were differentially expressed and their relative expression levels (FPKM value) in two groups (three biological replicates of each treatment) were shown as heat maps. H—high-temperature treatment for 24 h; TreH—trehalose pretreatment for 3 d followed by high-temperature treatment for 24 h. Abbreviations: PAL—phenylalanine ammonia lyase; CYP73A—trans-cinnamate 4-monooxygenase; 4CL—4-coumaric acid-CoA ligase; CHS—chalcone synthase; CHI—chalcone isomerase.

Exogenous trehalose also significantly affected the phenylpropanoid biosynthesis pathway at the high temperature, resulting in the accumulation of lignin and flavonoids (Figure 12A). Transcriptome data and metabolite profile analysis showed that most DEGs and a few metabolites, such as chalcone synthase (CHS), 4-coumaric acid-CoA ligase (4CL) and other key enzyme genes, caffeoyl quinic acid, coniferin, and other metabolites were upregulated during phenylpropanoid biosynthesis (Figure 12A). Flavonoids, such as luteolin and 2′-hydroxyisoflavones, were also significantly increased in the trehalose response network, which indicated that the regulation of phenylpropanoids and flavonoids played an important role in improving the heat tolerance of wheat in response to exogenous trehalose.

In Yannong 19, DEGs were also significantly enriched in starch and sucrose metabolic pathways, but most genes showed opposite expression patterns to those in Yangmai 18. Similarly, the expression levels of genes related to the TCA cycle remained upregulated (Figure 11B), which indicated that trehalose could affect the energy supply of these two wheat varieties. More amino acids and their derivatives (such as serine and pyroglutamic acid) were upregulated by exogenous trehalose. Similar to Yangmai 18, the purine metabolic pathway in Yannong 19 also showed more upregulation of genes related to guanine, xanthine and adenosine synthesis. The differences between the two varieties were that most of the genes in the metabolic pathway of glutamine-thiamine metabolism in Yannong 19 were downregulated (Supplementary Figure S3B).

DEGs in Yannong 19 were also significantly enriched in the phenylpropanoid biosynthesis pathway (Figure 12B), and the influencing genes were mainly related to lignin synthesis, but the expression pattern was opposite to that of Yangmai 18, which might be caused by variety differences.

## 3. Discussion

From the phenotypic results, it can be seen that exogenous trehalose improves the tolerance of plants to high temperature to a certain extent and reduces the degree of leaf wilting and yellowing (Figure 1), which may be because trehalose can protect the membrane system (cell membrane, chloroplast membrane and thylakoid structure) at high temperatures (Figure 3) and reduce the degree of cell membrane damage (Figure 2). This is similar to a previous study showing that trehalose could improve plant heat tolerance by maintaining biomembrane and protein function [11].

T6P, a precursor of trehalose, can be used as a signal to respond to carbon availability and regulate plant growth [15]. The metabolomics data show that exogenous trehalose did increase the T6P content in wheat, but the trehalose content was only significantly increased in Yangmai 18 (Figure 11A). The increase in trehalose content was accompanied by the upregulation of the trehalase gene and an increase in glucose content (Figure 11A). In Yannong 19, the trehalose content remained unchanged (Figure 11B), which was consistent with the view of some studies that trehalose accumulation is too low to play an osmotic adjustment role [14].

In the trehalose pretreatment group under high temperature, the two varieties of wheat showed different gene enrichment patterns. In Yangmai 18, the DEGs induced by trehalose were mainly related to photosynthesis and light reactions, while metabolism was more inclined to the synthesis of secondary metabolites, which may explain our observation of chloroplast structure (Figure 3) and the enrichment of secondary metabolites in the metabolome (Figure 10). In Yannong 19, the DEGs induced by trehalose were mainly related to alcohol metabolic processes, carboxylic acid transport and circadian rhythms, while the KEGG pathway was biased toward biosynthesis of amino acids and the phosphatidylinositol signaling system, suggesting that trehalose affects some signaling pathways.

From the results of integrated analysis, it was found that the expression of TCA cycle related genes in both wheat varieties was significantly upregulated (Supplementary Figure S2). TCA not only plays an important role in cell energy metabolism but also provides essential precursors for respiration, amino acid biosynthesis and general nitrogen metabolism. In salt-tolerant plants, TCA cycle activity and relevant metabolite transport can be altered to promote efficient respiration by making appropriate use of carbon sources, thus providing the energy to resist salt tolerance in salt-laden tissues [19]. Therefore, exogenous trehalose may promote the TCA cycle to provide more energy for high-temperature tolerance and provide precursors for generating stress-resistant substances to help wheat resist high temperatures. In addition, abiotic stress passively inhibits plant growth with cellular damage and/or limited resources (such as carbon dioxide, nutrients and energy). It is believed that breaking the growth-stress trade-off and rebalancing it may help plant growth under stress. We hypothesize that TCA activation helps plants make better use of energy, thus reducing the impact of heat stress on growth.

Trehalose pretreatment at high temperature will lead to reprogramming of wheat metabolism, including both primary and secondary metabolism. In primary metabolism, wheat accumulates a large amount of amino acids and their derivatives, such as proline, lysine, and glutamine (Figure 11). Proline can be used as an osmotic protectant and molecular chaperone and plays an important role in inhibiting ROS, stabilizing protein structure and maintaining intracellular homeostasis [20]. After trehalose pretreatment, the proline content in wheat increased to resist high temperature (Figure 11). The synthesis of phenylalanine is beneficial to the production of secondary metabolites, such as flavonoids. The results showed that genes related to phenylalanine metabolism were significantly upregulated by trehalose (Supplementary Figure S4). In addition to amino acids, purine metabolism in wheat is also significantly affected. It has been reported that some purine metabolites contribute to the stress protection in plants; for example, allantoin can synergistically activate ABA metabolism to enhance abiotic stress resistance [21]. The results of this study showed that the xanthine dehydrogenase (XDH) gene was upregulated (Supplementary Figure S3) in both wheat varieties. Studies have shown that XDH can remove H_2_O_2_ from chloroplasts under stress in Arabidopsis [22] and delay the senescence of rice (*Oryza sativa* L.) leaves [23]. Similarly, in wheat, XDH may play a similar role in stress resistance under the influence of trehalose. In addition, adenosine synthesis in the two wheat varieties was also promoted (Supplementary Figure S3). Adenosine is an important intermediate used in the synthesis of adenosine triphosphate (ATP), 3′,5′-cyclic ATP, adenine and adenylic acid. Sukrong et al. [24] found that adenine might regulate plant growth and stress resistance. According to the results, it is speculated that adenosine metabolism is involved in trehalose—induced resistance to high temperature, but further detection of related enzyme activities is needed.

After trehalose pretreatment, the changes in secondary metabolism mainly focused on the changes in the synthetic pathway of phenylpropanoids. In Yangmai 18, most DEGs and some of the metabolites, such as caffeoyl quinic acid and coniferin, were upregulated during phenylpropanoid biosynthesis (Supplementary Figure S4A). In addition, more lignin synthase genes were also upregulated by trehalose (Supplementary Figure S4A), such as genes of CHS and 4CL. The promotion of lignin synthesis may help plants to increase the thickness and strength of cell walls and reduce the wilting degree of plants. However, most of the related genes in Yannong 19 showed a downward trend, while lignin-related metabolites did not decrease significantly, which may be due to the difference in stress resistance mechanisms caused by different varieties (Supplementary Figure S4B). Lignin is an important component of the cell wall, and many studies have shown that lignin synthesis can help plants to resist salt [25] and drought [26]. Cell wall metabolism can be changed by high temperature, which is an important physiological mechanism to protect plants [27]. Rice seedlings grown under different high temperatures caused the differential expression of proteins related to lignin biosynthesis, including cell wall peroxidases (PRX) and phenylalanine ammonia lyase (PAL) [28]. In strawberry (*Fragaria* x *ananassa* cv. Camarosa), an increasing PRX activity was also detected under high temperature [29]. Trehalose may provide support for wheat to resist high temperature through the functional regulation of lignin biosynthesis.

These results also showed that the two wheat varieties had diverse resistances to high temperature and different responses to trehalose at high temperature. Under high-temperature stress conditions, Yannong 19 suffered less membrane damage than Yangmai 18 (Figure 2 and Figure 3), which indicated that Yannong 19 had stronger heat resistance. The statistical quantities of differential genes and metabolites also indicate that Yangmai 18 is more affected by exogenous trehalose than Yannong 19 (Figure 5). Combined analysis showed that the regulation of the TCA cycle and purine metabolism had the same trends in both varieties (Figure 11 and Figure S3). The difference between Yangmai 18 and Yannong 19 is that the gene metabolism regulation of Yangmai 18 is more focused on carbon metabolism (mainly sugar metabolism and signal transduction) and is part of flavonoid metabolism, upregulating the synthesis of more amino acids and their derivatives, and generating more protective substances (such as proline and cysteine) under stress (Figure 11A). According to reports by Buer et al. [30], flavonoids can act as antioxidants, combined with phytotoxins, to regulate the transport of auxin in the stress response, thereby affecting plant growth and development and resistance to oxidative stress caused by high temperature. However, the gene metabolite regulation in Yannong 19 is more concentrated on the biosynthesis of secondary metabolites, mainly involving the regulation of phenylpropanoid biosynthesis (Figure 12B and Figure S4).

**Figure 13:**
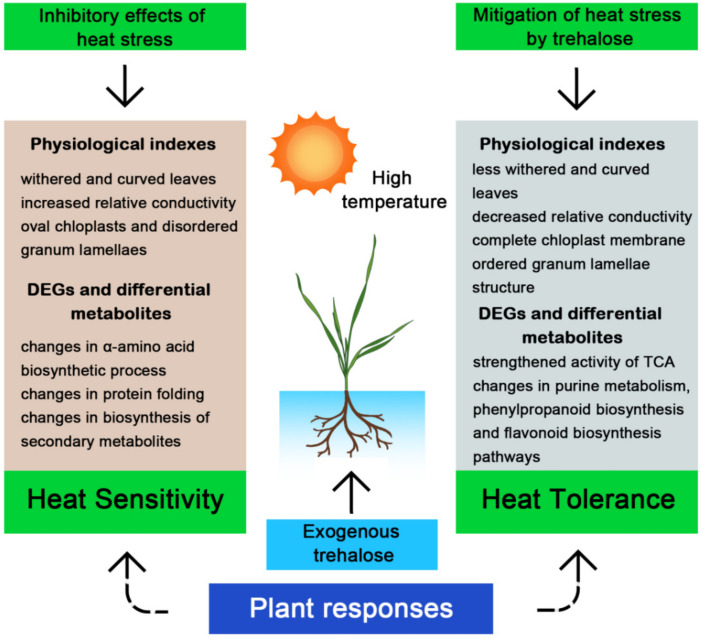
A proposed model of the mechanism of exogenous trehalose to alleviate the negative effect on wheat seedlings under high temperature.

Overall, exogenous trehalose can effectively enhance heat tolerance in wheat seedlings. The underlying mechanism of its mitigation of high-temperature damage was shown in Figure 13.

In addition, the genes and metabolites of the two varieties were compared in this study. The DEGs of the two varieties were mainly concentrated in ribosome, plant hormone signal transduction and oxidative phosphorylation (Supplementary Figure S5A). Differential metabolites focused on the biosynthesis of secondary metabolites, purine metabolism and the biosynthesis of flavone and flavonol (Supplementary Figure S5B). These findings explain why the lignin-synthesis mechanisms of Yangmai 18 and Yannong 19 showed different changes. Because of the different functions of flavone and flavonol biosynthesis, the effects brought by trehalose will also be different. However, it cannot be concluded that trehalose does not act on the phenylpropanoid synthesis pathway in Yannong 19 to achieve stress resistance, which needs further verification.

## 4. Materials and Methods

### 4.1. Plant Materials and Stress Treatment

Wheat (*T. Aestivum* L.) strains Yangmai 18 (spring wheat, Lixiahe, Jiangsu, China) and Yannong 19 (winter wheat, Yantai, Shandong, China) were used in this experiment. The wheat seeds were washed with water 2–3 times and soaked in a beaker for 24 h. After pouring out the water, a wet cloth was placed on the beaker which was then left to stand at room temperature for 24 h. Then well-germinated seeds were selected and placed in a plastic train with a special leakage net in an appropriate amount of water for cultivation (20 °C, light intensity 120 μmol m^−2^ s^−1^, light/dark 13/11 h), and water was changed every day. When the first leaf was fully expanded, the wheat seedlings were cultivated in Hoagland nutrient solution (the nutrient solution was changed every day). When the second leaf was completely unfolded, wheat seedlings were divided into 8 groups (with 15 plants in each group, with three replications for each group) for treatment (Table 3). The wheat seedlings cultured in nutrient solution without trehalose were named as control (CK), while those cultured in nutrient solution with 0.5 mM trehalose for 3 days were named as Tre treatments (Tre). The 0.5 mM trehalose-treated and untreated wheat seedlings were cultured in a light incubator at 42 °C for 24 h (100% light intensity, light/dark 13/11 h) represented with H and TreH respectively (Table 1). After heat stress, the second extended leaf of each treatment group was harvested for the determination of physiological parameters, the ultrastructure, as well as transcriptome and metabolome analyses. While the two groups (H and TreH) were treated at high temperature, the other groups (CK and Tre) were cultured at 20 °C for 24 h, and the second leaf of each group was used for the same follow-up analyses.

### 4.2. Phenotypic Analysis

A total of 10 plants were randomly selected from each treatment group for taking photos and calculating the ratio of withered and curved leaves. All the photos were taken soon after the high-temperature groups finishing the heat stress treatment. The ratio of withered leaves was defined as the proportion of the withered leaves in all leaves of the 10 plants per group (same for the ratio of curved leaves). The lengths of withered parts in leaves were measured by a ruler.

### 4.3. Physiological Measurement of Wheat Seedlings under High Temperature Stress

The 20 leaf discs from each treatment were placed in 10 mL of deionized water in a tube. Pump until the blades became transparent and sank underwater. After oscillating the tube at room temperature for 1 h, the initial conductivity value (S1) was measured by conductivity meter (Mettler Toledo, Greifensee, ZÜRICH, Switzerland). Subsequently, they were put in a boiling water bath for 5 min and then were cooled down to room temperature, and the final conductivity was measured (S2).

Relevant calculation formula:

$$\mathrm{Relative}\;\mathrm{conductivity}\;\left(L\right)=\frac{S_{1}}{S_{2}}\;\%$$

$$\mathrm{Relative}\;\mathrm{Electrolyte}\;\mathrm{leakage}\;\left(\mathrm{EL}\right)\;\left(\%\right)=\frac{L_{t}-L_{\mathrm{CK}}}{1-L_{\mathrm{CK}}}\times 100$$

L_t_—Relative conductivity of treated leaves;

L_CK_—Relative conductivity of control leaves.

### 4.4. Ultrastructure of Leaves

We followed the method of Gielwanowska et al. [31], with minor modifications. Fragments of the second leaf, about 2–3 mm in length, were collected from the control and treated groups. After the fixation, dehydration and embedment, ultra-thin sections were examined and photographs were taken with a JEM-2100 transmission electron microscope (JEOL Ltd., Musashino, Tokyo, Japan). A total of 5–6 biological replicates were selected for each treatment. The damaged chloroplasts ratio was defined as the proportion of the chloroplasts with a broken membrane or a deformed granum lamellae structure in all chloroplasts by analyzing 10 micrographs per group. The width-to-length ratio was calculated using the software Image J (National Institutes of Health, Bethesda, MD, USA), by analyzing average 10 chloroplasts per group.

### 4.5. RNA Isolation, cDNA Library Construction, and Sequencing

Total RNA was extracted from snap-frozen leaf samples. RNA concentration was measured with a Qubit RNA Assay Kit in a Qubit 2.0 Fluorometer (Life Technologies, Carlsbad, CA, USA). The integrity of RNA was evaluated by utilizing the RNA Nano 6000 Assay Kit of the Bioanalyzer 2100 system (Agilent Technologies, Santa Clara, CA, USA). The sequencing libraries preparation and high-throughput sequencing were done by Metware Biotechnology Co., Ltd. (Wuhan, Hubei, China, www.metware.cn, accessed on 1 September 2020). A total of 24 samples were sequenced and 218.39 Gb of Clean Data were obtained.

### 4.6. Analyses of DEGs, KEGG and GO

DE-seq2 [32] was performed to analyze differential expression between sample groups. Reads counting of genes was realized using featureCounts [33]. After the difference analysis, the Benjamini–Hochberg method was used to correct the *p* value by multiple hypothesis tests, and the false discovery rate was obtained. The screening conditions for differential genes were log_2_ Fold Change ≥ 1 and FDR < 0.05. Pathway significant enrichment analysis was based on the pathway in the KEGG database. The pathway that is significantly enriched in DEGs compared with the genome-wide background is found through hypergeometric test, and the result of KEGG enrichment is presented in the form of a scatter plot. The GO Term significant enrichment analysis was based on the GO Term in the GO database, and the hypergeometric test was used to find out GO Terms that were significantly enriched in DEGs compared with the whole genome background. The enrichment analysis results are presented in the form of bar chart.

### 4.7. Metabolite Extraction and Ultra Performance Liquid Chromatography–Tandem Mass Spectrometry (UPLC-MS/MS) Analysis

The sample was freeze-dried and grounded into powder, 100 mg of which was dissolved in 1.2 mL of 70% methanol. The dissolved samples were refrigerated at 4 °C overnight. To improve the extraction rate, the samples were vortexed 6 times during this period. Then the samples were centrifuged (rotation speed: 12,000 rpm, 10 min) at 4 °C, and finally the supernatant was filtered through a microporous membrane (pore size 0.22 μm) for UPLC-MS/MS analysis.

Liquid phase conditions mainly include:

(1) Column: Agilent SB-C18 1.8 µm, 2.1 mm × 100 mm;
(2) Mobile phase: Phase A is ultra-pure water (0.1% formic acid added), phase B is acetonitrile (0.1% formic acid added);
(3) Elution gradient: the proportion of phase B was 5% for 0.00 min, and the proportion of phase B linearly increased to 95% within 9.00 min, and maintained at 95% for 1min, 10.00–11.10 min, the proportion of phase B decreased to 5%, and was balanced at 5% for 14 min;
(4) Flow rate 0.35 mL/min; Column temperature 40 °C; injection volume 4 μL.

See Supplementary Table S1 for a list of metabolites. See Supplementary Figure S1 for the total ions current and the extracted ions current of mixed quality control samples.

Based on the results of orthogonal partial least squares-discriminant analysis (OPLS-DA), VIP of the multivariate analysis OPLS-DA model was used to screen the metabolites of different varieties or tissues. Meanwhile, combined with the *p*-value or FC of univariate analysis, the differential metabolites were further screened out. Screening criteria 1: Select metabolites with FC ≥ 1.5 or FC ≤ 0.67. If the FC of the metabolites in the control group and the treated group are greater than 1.5 times or less than 0.67, then the difference is regarded as significant. Criteria 2: If there is biological duplication in sample grouping, on the basis of the above, select metabolites with VIP ≥ 1. According to the results, KEGG pathway enrichment was carried out. After hypergeometric distribution calculation, KEGG enrichment results were presented in a scatter plot.

### 4.8. RT-qPCR Analysis

Wheat total RNA was extracted with UNIQ-10 column Trizol total RNA extraction kit (Sangon Biotech, Shanghai, China), cDNA was synthesized with two-step RT-qPCR premix (genomic removal) kit (Vazyme Biotech, Nanjing, Jiangsu, China), and RT-qPCR reaction system was prepared with universal high-sensitivity dye quantitative PCR detection kit (Vazyme Biotech, Nanjing, Jiangsu, China). Using a fluorescence quantitative PCR instrument (BIORAD CFX-96, BIORAD, Hercules, CA, USA), the reaction program was run, and the relative expression of each gene was calculated by $2^{-\Delta \Delta \mathrm{CT}}$ method. See Supplementary Table S2 for a list of primers.

### 4.9. Statistical Analysis

A quantitative assessment was conducted on randomly selected samples from three independent biological replicates. Two-way ANOVA and unpaired *t*-test were used to evaluate the significant differences in EL among the experimental groups. GraphPad Prism (8.2.1, GraphPad Software, San Diego, CA, USA) was used for plotting.

## 5. Conclusions

In this study, the changes in mRNA sequence and metabolite abundance in leaves of two varieties of wheat after trehalose pretreatment at high temperature were comprehensively analyzed. The results showed that trehalose protected the wheat biomembrane system under heat stress, promoted carbohydrate metabolism and signal transduction, enhanced TCA cycle activity, and regulated purine metabolism, gene expression and metabolite accumulation in the phenylpropanoid biosynthesis and flavonoid biosynthesis pathways to improve high temperature resistance. These findings can provide specific directions for further understanding the mechanism of trehalose to help plants to resist stress and provide a scientific theory foundation for the practical application of trehalose in later periods.

## Figures and Tables

**Table 1:** Differential gene statistics.

Group	Total	Down	Up
T_I_CK_vs_T_I_H	29,886	14,200	15,686
T_I_CK_vs_T_I_Tre	3158	1623	1535
T_I_H_vs_T_I_TreH	18,352	9920	8432
T_I_Tre_vs_T_I_TreH	26,720	12,357	14,363
T_II_CK_vs_T_II_H	24,791	11,438	13,353
T_II_CK_vs_T_II_Tre	1286	364	922
T_II_H_vs_T_II_TreH	9045	4901	4144
T_II_Tre_vs_T_II_TreH	20,075	9772	10,303

T—the transcriptome; Ⅰ—Yangmai 18; Ⅱ—Yannong 19; CK—control group; H—heat stress treatment for 24 h; Tre—trehalose pretreatment for 3 d; TreH—trehalose pretreatment for 3 d followed by high-temperature treatment for 24 h.

**Table 2:** Differential metabolite statistics.

Group	Total	Down	Up
M_I_CK_vs_M_I_H	256	113	143
M_I_CK_vs_M_I_Tre	53	34	19
M_I_H_vs_M_I_TreH	183	96	87
M_I_Tre_vs_M_I_TreH	248	109	139
M_II_CK_vs_M_II_H	218	105	113
M_II_CK_vs_M_II_Tre	117	65	52
M_II_H_vs_M_II_TreH	77	50	27
M_II_Tre_vs_M_II_TreH	240	123	117

M—the metabolomics; I—Yangmai 18; II—Yannong 19; CK—control group; H—heat stress treatment for 24 h; Tre—trehalose pretreatment for 3 d; TreH—trehalose pretreatment for 3 d followed by high-temperature treatment for 24 h.

**Table 3:** Treatment of each experimental group of wheat.

Group Name	Pretreatment with 0.5 mM Trehalose (Tre)	High-Temperature Stress Treatment (H)
Ⅰ_CK		
Ⅰ_Tre	✓	
Ⅰ_H		✓
Ⅰ_TreH	✓	✓
Ⅱ_CK		
Ⅱ_Tre	✓	
Ⅱ_H		✓
Ⅱ_TreH	✓	✓

I—Yangmai 18; II—Yannong 19; CK—control group; H—heat stress treatment for 24 h; Tre—trehalose pretreatment for 3 d; TreH—trehalose pretreatment for 3 d followed by high-temperature treatment for 24 h.

## Data Availability

The datasets for this study can be found in the National Center for Biotechnology Information (NCBI) repository, bioproject: PRJNA787788.

## Supplementary Materials

The following are available online at https://www.mdpi.com/article/10.3390/ijms23095194/s1.
