# Supplementary material for: Transcriptomic and Metabolomic Analyses of the Effects of Exogenous Trehalose on Heat Tolerance in Wheat
Source: Int J Mol Sci. 2022 May 6;23(9):5194. doi: 10.3390/ijms23095194 (PMC9103215; doi:10.3390/ijms23095194)
Supplement: Supplementary file 1 [file ijms-23-05194-s001.zip › Figure S1 Qualitative analysis of metabolites.pdf]

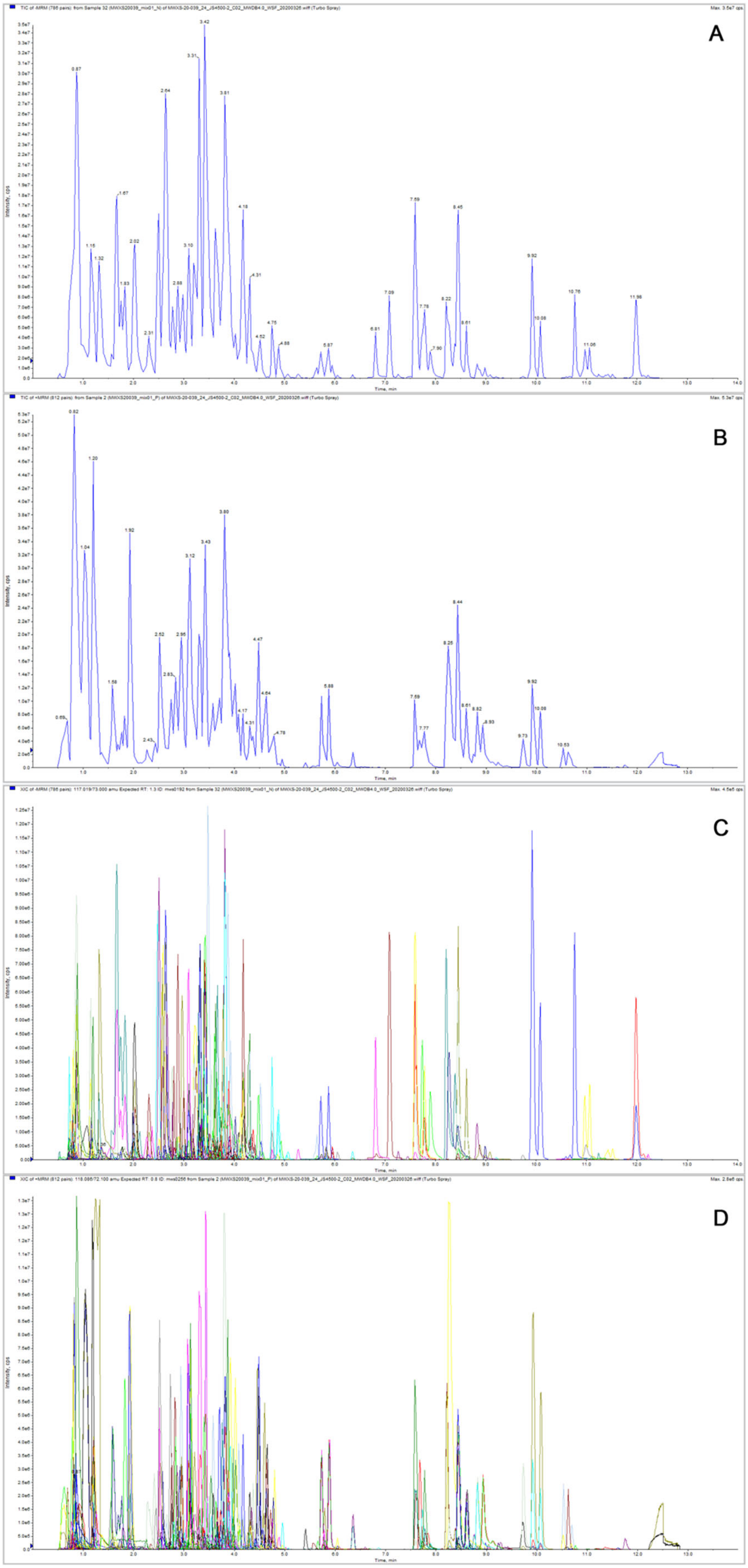
A
B
C
D
Intensity, cps
Time, min

Supplementary Figure S1. Qualitative analysis of metabolites. (A) (B) Total ions current of mixed quality control samples. **(A)** Negative ion mode. **(B)** Positive ion mode. **(C) (D)** Extracted ions current of mixed quality control samples. **(C)** Negative ion mode. **(D)** Positive ion mode. Abscissa represents the retention time (Rt) of the metabolite detection, and ordinate represents the ion flow intensity of the detection (unit: count per second). The area of each chromatographic peak represents the relative content of the corresponding substance.
