# Supplementary material for: Transcriptomic and Metabolomic Analyses of the Effects of Exogenous Trehalose on Heat Tolerance in Wheat
Source: Int J Mol Sci. 2022 May 6;23(9):5194. doi: 10.3390/ijms23095194 (PMC9103215; doi:10.3390/ijms23095194)
Supplement: Supplementary file 1 [file ijms-23-05194-s001.zip › Figure S4 Gene-metabolite integrated analysis pathway diagram of phenylpropanoid biosynthesis.pdf]

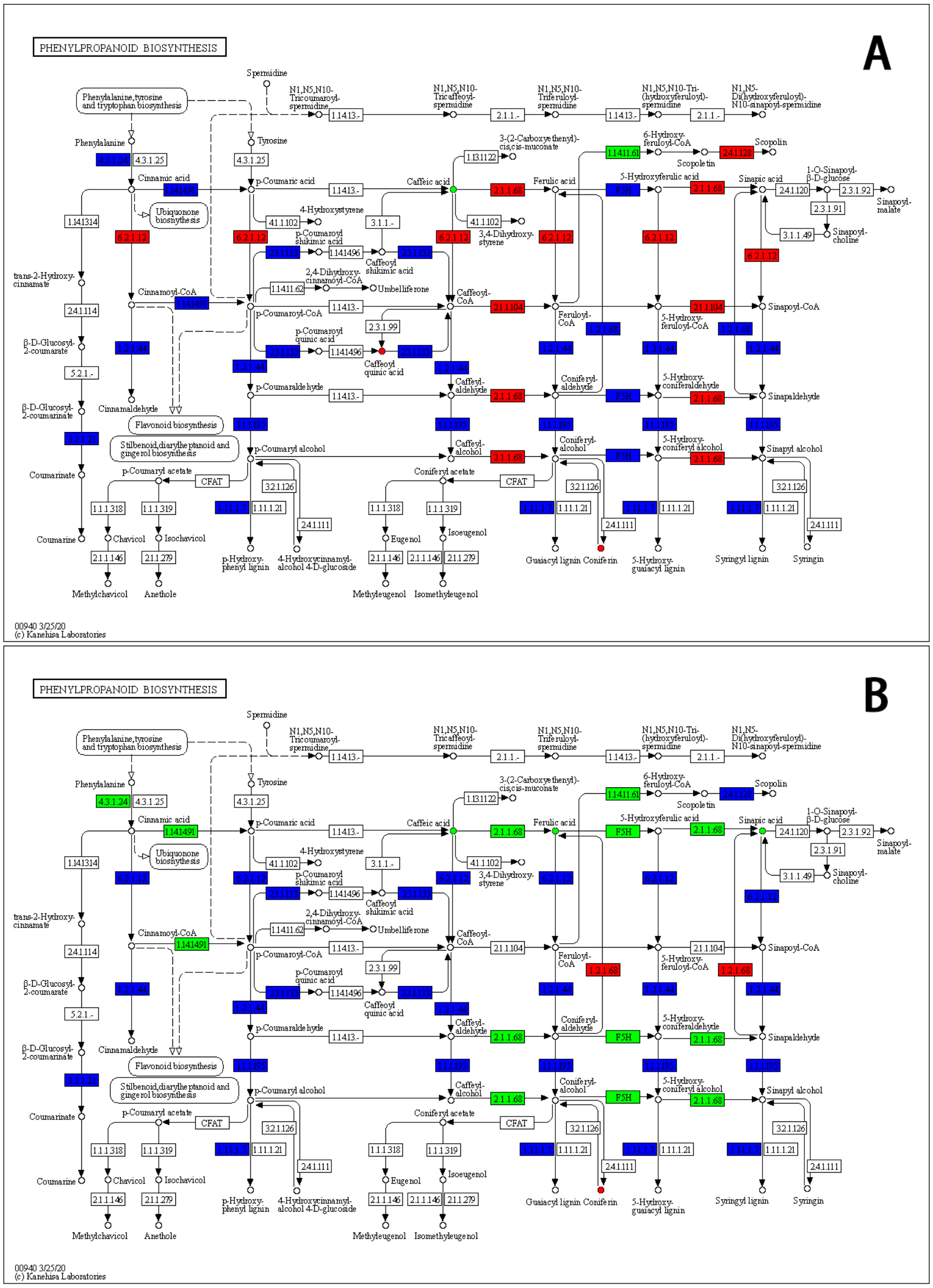

Supplementary Figure S4. Gene-metabolite integrated analysis pathway diagram of phenylpropanoid biosynthesis. (A) Yangmai 18. **(B)** Yannong 19. The red boxes represent genes that are up regulated, the green boxes represent genes that are down regulated, and the blue boxes represent genes that are partially up regulated and partially down regulated. The red circles represent metabolites that are up regulated. The green circles represent metabolites that are down regulated.
