# Supplementary material for: Transcriptomic and Metabolomic Analyses of the Effects of Exogenous Trehalose on Heat Tolerance in Wheat
Source: Int J Mol Sci. 2022 May 6;23(9):5194. doi: 10.3390/ijms23095194 (PMC9103215; doi:10.3390/ijms23095194)
Supplement: Supplementary file 1 [file ijms-23-05194-s001.zip › Figure S5 KEGG metabolic pathway enrichment of DEGs and differential metabolites of two kinds of wheat without trehalose pretreatment at high temperature.pdf]

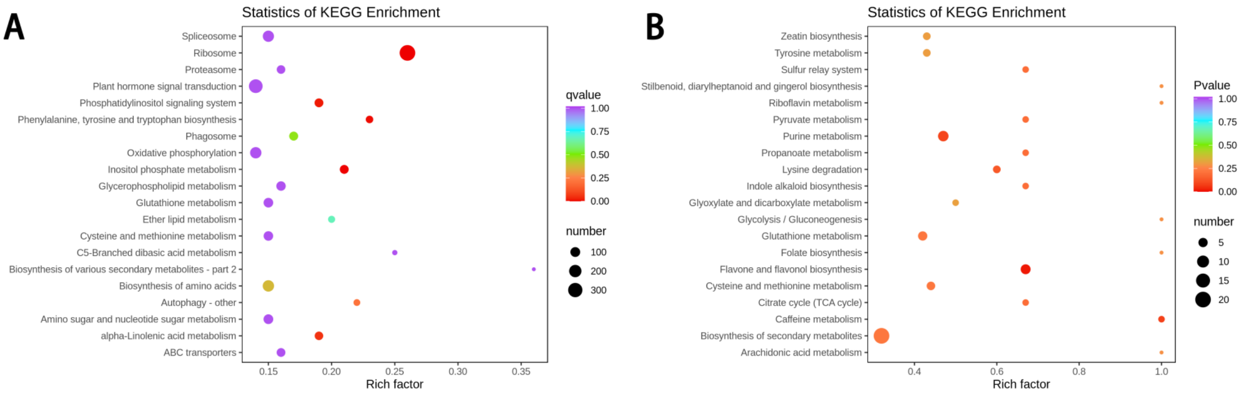

Supplementary Figure S5. KEGG metabolic pathway enrichment of DEGs and differential metabolites of two kinds of wheat without trehalose pretreatment at high temperature. (A) Transcriptome. **(B)** Metabonomics. The ordinate represents the KEGG pathway. The abscissa represents the Rich factor. The larger the rich factor, the greater the enrichment degree. The larger the dot, the more the number of differential genes enriched by the pathway. The redder the color of the dot, the more significant the enrichment.
