# Supplementary material for: Transcriptomic and Metabolomic Analyses of the Effects of Exogenous Trehalose on Heat Tolerance in Wheat
Source: Int J Mol Sci. 2022 May 6;23(9):5194. doi: 10.3390/ijms23095194 (PMC9103215; doi:10.3390/ijms23095194)
Supplement: Supplementary file 1 [file ijms-23-05194-s001.zip › Table S2 Primer List.pdf]

Supplementary Table S2. Primer List

Gene	Sequence (5 'to 3')	
*Ubiquitin**	AGATCCAGGACAAGGAGGGT	F
	GGAGCACCAAGTGGAGAGTAG	R
RAV	CTTCCCTCTTGGCGGCGACTC	F
	GCACGTAGCTCTGGCTGCTCG	R
NPR1	TCGAGCAGCTCGCCTTGCTGG	F
	CGTGCGACGAGGTGGGAGCAG	R
USP36_42	TCGTCGTTCCGCCGCCCATACC	F
	GAGGCCGAGCAGTCCACCGAG	R
COX17	CGGCTGCTTGCTCCATTGTGA	F
	CACTTGGTGCACGCAGACTCC	R
BAG1	AGCGGGGAGATCGAGTGGGAG	F
	GTGGCCACCTGACCGTGATG	R
OPR	AGATCCACGGCGCACACGGTT	F
	GCGGCACCAGTTCTCGAGGCT	R
NRT	CTCCTTCGGCAACCCACACAT	F

	CCATAACGTGGGCCAAGGAGG	R
SPOP	GCGGAGGTCGCCGACTACGTG	F
	CCGGCGAGGCGGATGTCGTAC	R
PEP4	GTACTCCGGCGTCATGGGGTGG	F
	GCACTGCACCCAGGACACGTC	R
ILKAP	GTCACCGCGCTTTCGTACTAC	F
	ACAGGCCTCCAATCAGCAGTA	R

* Indicates an internal reference gene.
