# Supplementary material for: Transcriptomic and Metabolomic Analyses of the Effects of Exogenous Trehalose on Heat Tolerance in Wheat
Source: Int J Mol Sci. 2022 May 6;23(9):5194. doi: 10.3390/ijms23095194 (PMC9103215; doi:10.3390/ijms23095194)
Supplement: Supplementary file 1 [file ijms-23-05194-s001.zip › Table S3 Transcriptome sequencing quality of Triticum aestivum L.pdf]

Supplementary Table S3. Transcriptome sequencing quality of *Triticum aestivum* L.

Sample	Raw Reads	Clean Reads	Q30(%)	GC Content (%)	Reads mapped	Unique mapped
T_II_Tre1	67353224	65255790	94.7	54.95	61824259(94.74%)	58598739(89.80%)
T_II_Tre2	76419692	75363154	94.76	55.8	71648824(95.07%)	67695514(89.83%)
T_II_Tre3	66532924	65073006	94.57	56.42	61882020(95.10%)	58396707(89.74%)
T_I_Tre1	54755446	53373362	94.93	55.22	51021787(95.59%)	48136382(90.19%)
T_I_Tre2	59245142	57929558	94.54	56.2	55479187(95.77%)	52327362(90.33%)
T_I_Tre3	62356842	60982284	94.75	55.51	58229262(95.49%)	55127443(90.40%)
T_II_TreH1	57709886	56601568	94.57	54.96	53734103(94.93%)	50916721(89.96%)
T_II_TreH2	66641204	65623710	94.53	54.17	62192086(94.77%)	59010637(89.92%)
T_II_TreH3	64983130	63295888	94.67	56.07	60027522(94.84%)	56757384(89.67%)
T_I_TreH1	58898388	57872988	94.68	54.37	55438879(95.79%)	52706715(91.07%)
T_I_TreH2	62638158	61514930	95.76	54.79	58875181(95.71%)	55845617(90.78%)
T_I_TreH3	65849702	64591712	95.67	54.39	61840877(95.74%)	58591997(90.71%)
T_II_CK1	56663836	55216592	94.45	52.87	51831111(93.87%)	49524806(89.69%)
T_II_CK2	63559602	61972300	94.45	55.02	58692986(94.71%)	55578732(89.68%)

T_II_CK3	52628036	51628352	94.76	54.32	48895520(94.71%)	46441036(89.95%)
T_I_CK1	55040632	53692606	94.64	52.52	50728763(94.48%)	48385437(90.12%)
T_I_CK2	68037142	66297176	94.32	55.37	63017745(95.05%)	59626519(89.94%)
T_I_CK3	47585656	46545960	94.85	55.38	44496518(95.60%)	41991891(90.22%)
T_II_H1	63434022	61899504	94.66	54.51	58507902(94.52%)	55404773(89.51%)
T_II_H2	73452672	72428548	94.41	54.96	68370658(94.40%)	64886034(89.59%)
T_II_H3	67362280	65785344	94.26	55.25	62022944(94.28%)	58830916(89.43%)
T_I_H1	53090664	51756132	94.67	55.84	48943436(94.57%)	46413948(89.68%)
T_I_H2	61661246	60504818	94.26	57.3	56382302(93.19%)	53241761(88.00%)
T_I_H3	61787236	60835646	94.67	55.63	57473624(94.47%)	54431932(89.47%)

T: the transcriptome; 1, 2, 3: repeating groups; Ⅰ: Yangmai 18; Ⅱ: Yannong 19; CK: control group; H: heat stress treatment for 24 h; Tre: trehalose pretreatment for 3 d; TreH: trehalose pretreatment for 3 d followed by high-temperature treatment for 24 h.
